# Influencing factors and prognostic value of left ventricular systolic dysfunction in patients with complete occlusion of the left anterior descending artery reperfused by primary percutaneous coronary intervention

**DOI:** 10.1186/s12872-023-03341-5

**Published:** 2023-07-10

**Authors:** Yongle Jing, Chengzhi Lu, Suzhen Guo, Bingwei Chen, Xuying Ye, Qiang He, Wei Xia, Ting Xin

**Affiliations:** 1 The First Central Clinical School, Tianjin Medical University, No 22 Qixiangtai Road, Tianjin, 300070 Heping District China; 2 Department of Cardiology, Tianjin First Central Hospital, No 24 Fukang Road, Tianjin, 300192 Nankai District China

**Keywords:** Acute anterior wall ST-segment elevation myocardial infarction, Left ventricular systolic dysfunction, Risk factors, Prognostic value

## Abstract

**Background:**

The aim of this study was to perform a retrospective analysis of patients with acute anterior wall ST-segment elevation myocardial infarction (AAW-STEMI) whose left anterior descending (LAD) artery was completely occluded and reperfused by primary percutaneous coronary intervention (PPCI) and to determine the influencing factors and prognostic value of left ventricular systolic dysfunction (LVSD) in the acute phase of acute myocardial infarction (AMI).

**Methods:**

A total of 304 patients with AAW-STEMI were selected. The selected patients were divided into two groups: the preserved left ventricular ejection fraction (pLVEF) group (LVEF ≥ 50%, n = 185) and the reduced left ventricular ejection fraction (rLVEF) group (LVEF < 50%, n = 119). The influencing factors of LVSD and their predictive value for LVSD were analyzed. Patients were followed up by examining outpatient records and via telephone. The predictive value of LVSD for the cardiovascular mortality of patients with AAW-STEMI was analyzed.

**Results:**

Age, heart rate (HR) at admission, number of ST-segment elevation leads (STELs), peak creatine kinase (CK) and symptom to wire-crossing (STW) time were independent risk factors for LVSD (*P* < 0.05). The receiver operating characteristic (ROC) analysis showed that the peak CK had the strongest predictive value for LVSD, with an area under the curve (AUC) of 0.742 (CI, 0.687 to 0.797) as the outcome. At a median follow-up of 47 months (interquartile range, 27 to 64 months), the Kaplan‒Meier survival curves up to 6-year follow-up revealed a total of 8 patients succumbed to cardiovascular disease, with 7 (6.54%) in the rLVEF group and 1 (0.56%) in the pLVEF group, respectively (hazard ratio: 12.11, [*P* = 0.02]). Univariate and multivariate Cox proportional hazards regression analysis demonstrated that rLVEF was an independent risk predictor of cardiovascular death in patients with AAW-STEMI discharged after PPCI (*P* < 0.01).

**Conclusions:**

Age, HR at admission, number of STELs, peak CK, and STW time may be used to identify patients with a high risk of heart failure (HF) in a timely manner and initiate early standard therapy for incident LVSD in the acute phase of AAW-STEMI reperfused by PPCI. A trend toward increased cardiovascular mortality at follow-up was significantly linked to LVSD.

## Introduction

New-onset heart dysfunction is a common complication of acute myocardial infarction (AMI). Acute anterior wall ST-segment elevation myocardial infarction (AAW-STEMI) leads to more pronounced left ventricular systolic dysfunction (LVSD) and more adverse left ventricular (LV) remodeling than STEMI in other areas [1]. Guidelines have been presented to support that the standard treatment for patients with STEMI is primary percutaneous coronary intervention (PPCI), modern antithrombotic therapy and secondary prevention [2, 3]. Despite rapid advances in pharmacological and interventional treatment strategies, STEMI remains the leading cause of heart failure (HF) and mortality. Evidence has shown that the incidence of HF in patients with STEMI after PPCI is 4.6% at 1 month, 4.7% at 1 year, and 5.1% at 2 years [4].

LVSD can be represented by left ventricular ejection fraction (LVEF), and it is defined as LVEF < 50% [5]. Reduced LVEF (rLVEF) is well known to be associated with high AMI mortality, and it is associated with increased cardiovascular risk and a higher rate of unplanned cardiac rehospitalizations in the long term [6, 7]. Improvement of LVEF after revascularization among STEMI patients is associated with improved long-term survival. Clinical practice guidelines have provided several therapeutic recommendations for both the acute and long-term post-discharge management of AMI based on LVEF, such as angiotensin converting-enzyme inhibitors (ACEIs), angiotensin receptor blockers (ARBs) and aldosterone antagonists [3]. The ongoing PARADISE-AMI trial is evaluating the safety and efficacy of angiotensin receptor II blocker-neprilysin inhibitors (ARNIs) compared with ramipril in addition to standard post myocardial infarction (MI) pharmacotherapy [8]. To further improve the outcome of AAW-STEMI patients, it is necessary to identify earlier and easily assessable predictors of poor clinical outcome. This study aimed to investigate patients whose left anterior descending (LAD) artery was completely occluded and reperfused by PPCI to determine the influencing factors of LVSD in the acute phase of AAW-STEMI and to analyze the clinical prognostic value of LVSD.

## Methods

### Study population

Adopting a retrospective cohort research method, a total of 547 patients with AAW-STEMI reperfused by PPCI were selected in the Tianjin First Central Hospital from January 2016 to September 2021. All selected patients fulfilled the diagnostic criteria of AAW-STEMI in the “2020 ESC Guidelines for the management of acute coronary syndromes in patients presenting without persistent ST-segment elevation “ [9]. PPCI was successfully performed to open the occluded LAD artery in a standard fashion by 2 experienced cardiologists. Thrombus aspiration, post balloon dilation, and glycoprotein IIb/IIIa receptor antagonists were given at the discretion of the physician performing the intervention. All patients were given standard drug therapy during the perioperative period of PCI. The baseline characteristics were recorded.

The inclusion criteria were as follows. (1) The symptom to admission time was within 12 h. (2) The cardiac function classification at admission was Killip I. (3) Emergency angiography confirmed complete occlusion of the LAD artery without other coronary occlusions. (4) The postprocedural thrombolysis in myocardial infarction (TIMI) grade was TIMI grade 3 flow.

The exclusion criteria were as follows. (1) Coronary angiography showing incomplete occlusion of the LAD artery. (2) Patients whose cardiac function was Killip grade 2 or above upon admission. (3) Coronary angiography showing occlusion of the left main artery. (4) The postprocedural TIMI grade was less than TIMI grade 3 flow. (5) History of old myocardial infarction or chronic heart failure. (6) Complicated with liver failure (CLIF Consortium Acute on Chronic Liver Failure score > 64) or renal failure (estimated glomerular filtration rate < 30 mL/min/1.73m^2^). (7) Patients who died during hospitalization. (8) Abandon treatment and discharge automatically.

**Fig. 1:**
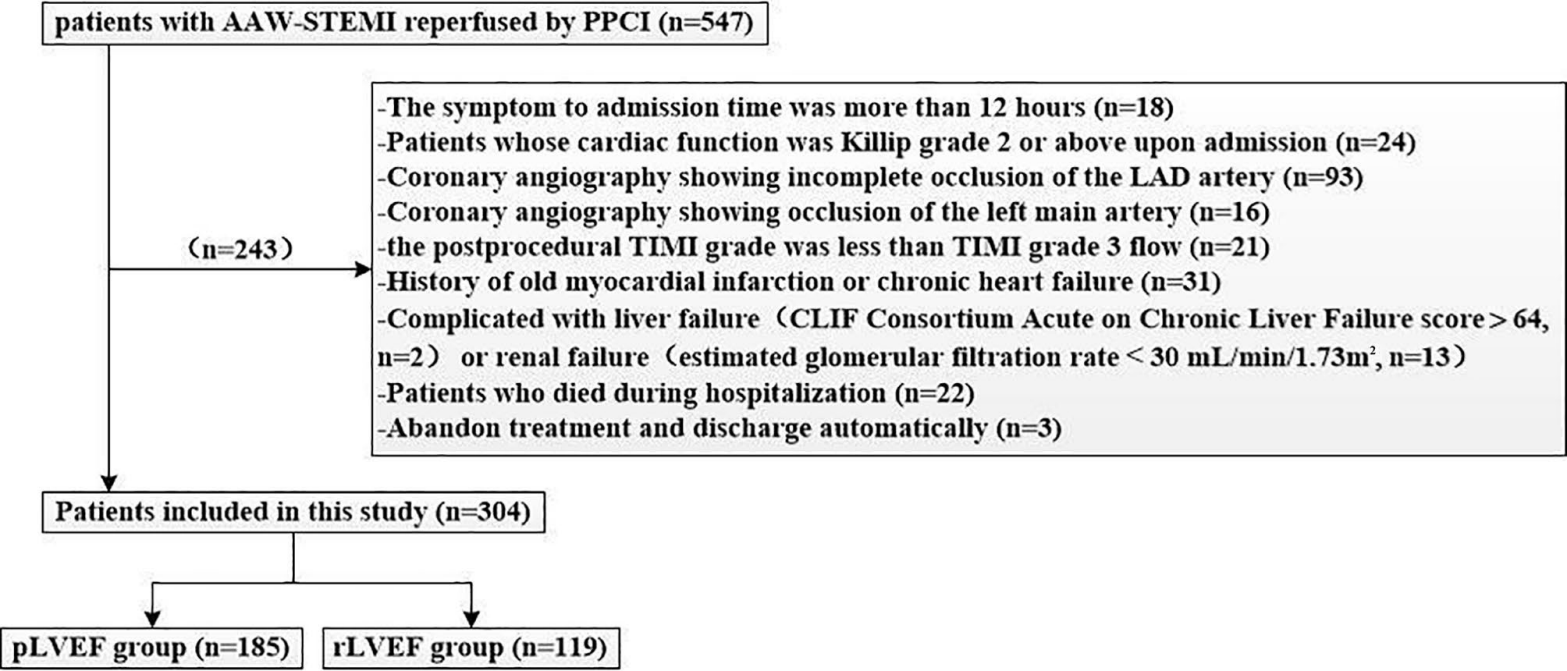
Study population

LVEF assessment by transthoracic echocardiography was preferably estimated using the Simpson biplane formula in PHLIP’S IE 33 equipment at 3–5 days after onset of STEMI. Finally, there were 304 patients meeting inclusion and exclusion criteria. They were divided into preserved LVEF (pLVEF) group (LVEF ≥ 50%) and rLVEF group (LVEF < 50%), see Fig. 1. Patients survived to hospital discharge were followed by telephone call or clinical visit for up to 6 years. The primary endpoint was cardiovascular death. Cardiovascular death was defined as the primary cause of death determined to be atherosclerotic cardiovascular disease, arrhythmia, heart failure, or sudden cardiovascular death.

### Statistical analysis

Continuous variables with a normal distribution are reported as the mean ± standard deviation ($\bar x \pm s$). Differences between two groups were analyzed by Student’s t test. Nonnormally distributed data are presented as the median and interquartile range (IQR). Differences between two groups were analyzed by the Mann‒Whitney U test. Categorical variables are reported as frequencies and percentages, and they were analyzed using the chi square test. The univariate logistic regression analysis was used to evaluate demographic, clinical, and biochemical variables with potential meaningful factors. After screening out variables with significant differences, multivariate logistic regression analysis was performed to test the independent influencing factors for LVSD. And the forward stepwise regression method was used to carry out multi-factor analysis. The receiver operating characteristic (ROC) curve was used to evaluate the predictive value of related risk factors for LVSD. The survival rate was determined via the Kaplan‒Meier test. The survival rate between the two groups was compared with the use of the log-rank statistic. The univariate Cox proportional hazards regression analysis was used to evaluate the variables with potential predictive value. The significantly different variables in univariate Cox regression analysis were included in multivariate Cox regression analysis. The Wald regression method was used to screen the predictors of cardiovascular death. A *P* value of < 0.05 was considered significant.

## Results

The mean age of the selected patients was 58.63 ± 11.81 years old (range 27–84 years), and 254 (83.55%) were male. Differences in the baseline and clinical outcomes between the pLVEF group and the rLVEF group are summarized in Table 1. There were statistically significant differences in age, prior ischemic stroke, STW time, HR at admission, ST-segment elevation leads (STELs) and their number, high-density lipoprotein (HDL) cholesterol levels, peak CK, N-terminal pro brain natriuretic peptide (NT-proBNP), proportion of LAD artery opening or proximal occlusion, occlusion site of LAD artery, prescription rates of Glycoprotein IIb/IIIa receptor antagonists and beta-blocker, and global registry of acute coronary events risk (GRACE) score between the two groups (*P* < 0.05).

**Table 1:** Baseline clinical characteristics stratified by LVEF assessment

	pLVEF group (n = 185)	rLVEF group (n = 119)	Univariate analysis	
			OR (95%C.I.)	*P*-value
Age (SD), years	56.96 ± 11.64	61.24 ± 11.67	1.032(1.011,1.054)	0.002
Men, n (%)	157 (84.86)	97 (81.51)	0.786(0.426,1.452)	0.442
Baseline risk factors				
Hypertension, n (%)	83 (44.86)	58 (48.74)	1.168(0.736,1.854)	0.508
Diabetes Mellitus, n (%)	43 (23.24)	25 (21.01)	0.878(0.503,1.534)	0.648
Smoking, n (%)	134 (72.43)	75 (63.03)	0.649(0.396,1.062)	0.084
Family history of CHD, n (%)	25 (13.51)	15 (12.61)	0.923(0.465,1.833)	0.819
Prior ischemic stroke, n (%)	17 (9.19)	25 (21.01)	2.628(1.351,5.115)	0.004
Anthropometric measurements				
SBP (SD), mmHg	138.98 ± 19.74	139.15 ± 24.27	1.000(0.990,1.011)	0.947
DBP (SD), mmHg	86.74 ± 14.42	89.02 ± 14.79	1.011(0.995,1.027)	0.181
HR (SD), bpm	75.09 ± 13.45	80.70 ± 14.00	1.030(1.012,1.048)	0.001
Manifestations of ECG				
Number of STELs [IQR]	5 (4, 6)	6 (5, 7)	1.457(1.253,1.694)	<0.001
STELs			1.178(1.078,1.287)	0.002
V1-V2, n (%)	3 (1.62)	0 (0)		-
V3-V4, n (%)	1 (0.54)	1 (0.84)		-
V1/2-V3/4, n (%)	72 (38.92)	20 (16.81)		<0.001
V1/2-V5/6, n (%)	46 (24.86)	36 (30.25)		0.302
V1/2-V3/4 +	13 (7.03)	10 (8.40)		0.658
I/aVL, n (%)				
V3/4-V6 + I/aVL, n (%)	2 (1.08)	1 (0.84)		-
V1/2-V5/6 +	48 (25.95)	51 (42.86)		0.002
I/aVL, n (%)				
Laboratory findings				
WBC [IQR],10^9^/L	10.20 (8.50,12.26)	10.02 (8.00,12.05)	0.960(0.894,1.030)	0.409
Creatinine (SD), umol/L	74.33 ± 16.55	72.97 ± 15.63	0.995(0.980,1.009)	0.475
TG [IQR], mmol/L	1.55 (1.05,2.66)	1.63 (1.07, 2.43)	0.898(0.773,1.044)	0.629
TC (SD), mmol/L	4.80 ± 1.04	4.94 ± 0.89	1.153(0.912,1.457)	0.234
HDL [IQR], mmol/L	1.04 (0.86,1.21)	0.93 (1.08,1.25)	2.498(1.051,5.938)	0.020
LDL (SD), mmol/L	3.26 ± 0.89	3.37 ± 0.80	1.159(0.885,1.518)	0.284
Blood glucose [IQR], mmol/L	7.51 (6.38,9.47)	7.78 (6.40, 9.75)	1.043(0.971,1.12)	0.314
NT-proBNP [IQR], ng/ml	52.10 (24.50,128.45)	83.00 (38.00,156.50)	1.000(1.000,1.001)	0.010
Peak CK [IQR], U/cL	26.19 (18.24,41.53)	45.11 (29.72,57.45)	1.047(1.032,1.062)	<0.001
Angiography results				
Occlusion site of LAD artery			0.532(0.358,0.789)	0.005
Opening or proximal, n (%)	83 (44.86)	74 (62.18)		0.003
Middle, n (%)	97 (52.43)	45 (37.82)		0.013
Distal, n (%)	5 (2.70)	0 (0)		-
Number of diseased vessels			1.330(0.998,1.773)	0.135
1 vessel, n (%)	56 (30.27)	27 (22.69)		0.148
2 vessels, n (%)	61 (32.97)	35 (29.41)		0.514
3 vessels, n (%)	68 (36.76)	57 (47.90)		0.054
FTW time [IQR], min	65.00 (54.00, 85.00)	72.00 (54.00,100.00)	0.006(0.999,1.012)	0.188
STW time [IQR], min	202.00 (131.50, 284.00)	254.00 (188.00, 394.00)	1.003(1.001,1.004)	<0.001
Thrombus aspiration, n (%)	31 (16.76)	12(10.08)	0.557(0.274,1.134)	0.103
post-balloon dilation, n (%)	96(51.89)	75(63.03)	1.580(0.987,2.531)	0.056
Complete revascularization, n (%)	35(18.92)	24(20.17)	1.083(0.606,1.933)	0.788
Medication use				
Glycoprotein IIb/IIIa receptor antagonists, n (%)	132(71.35)	98 (82.35)	1.874(1.061,3.310)	0.029
ACEIs/ARBs, or ARNIs, n (%)	125 (67.57)	82 (68.91)	1.089(0.720,1.647)	0.881
Beta-blocker, n (%)	126 (68.11)	95 (79.83)	1.854(1.076,3.194)	0.025
LVEF [IQR], %	54.00 (50.00,56.00)	45.00 (42.00,47.00)		<0.001
GRACE score	130.32 ± 21.96	138.13 ± 22.78	1.016(1.005,1.027)	0.004

SD: standard deviation, IQR: interquartile range, CHD: coronary heart disease, SBP: systolic blood pressure, DBP: diastolic blood pressure, HR: heart rate, bpm: beats per minute, ECG: electrocardiogram, STELs: ST-segment elevation leads, WBC: white blood cell, TG: triglyceride, TC: total cholesterol, HDL: high density lipoprotein, LDL: low density lipoprotein, NT-proBNP: N-terminal pro brain natriuretic peptide, CK: creatine kinase, LAD: left anterior descending artery, FTW: first medical contact to wire-crossing, STW: symptom to wire-crossing, ACEIs: angiotensin-converting enzyme inhibitors, ARBs: angiotensin receptor blockers, ARNIs: angiotensin receptor II blocker-neprilysin inhibitors, LVEF: left ventricular ejection fraction

**Fig. 2:**
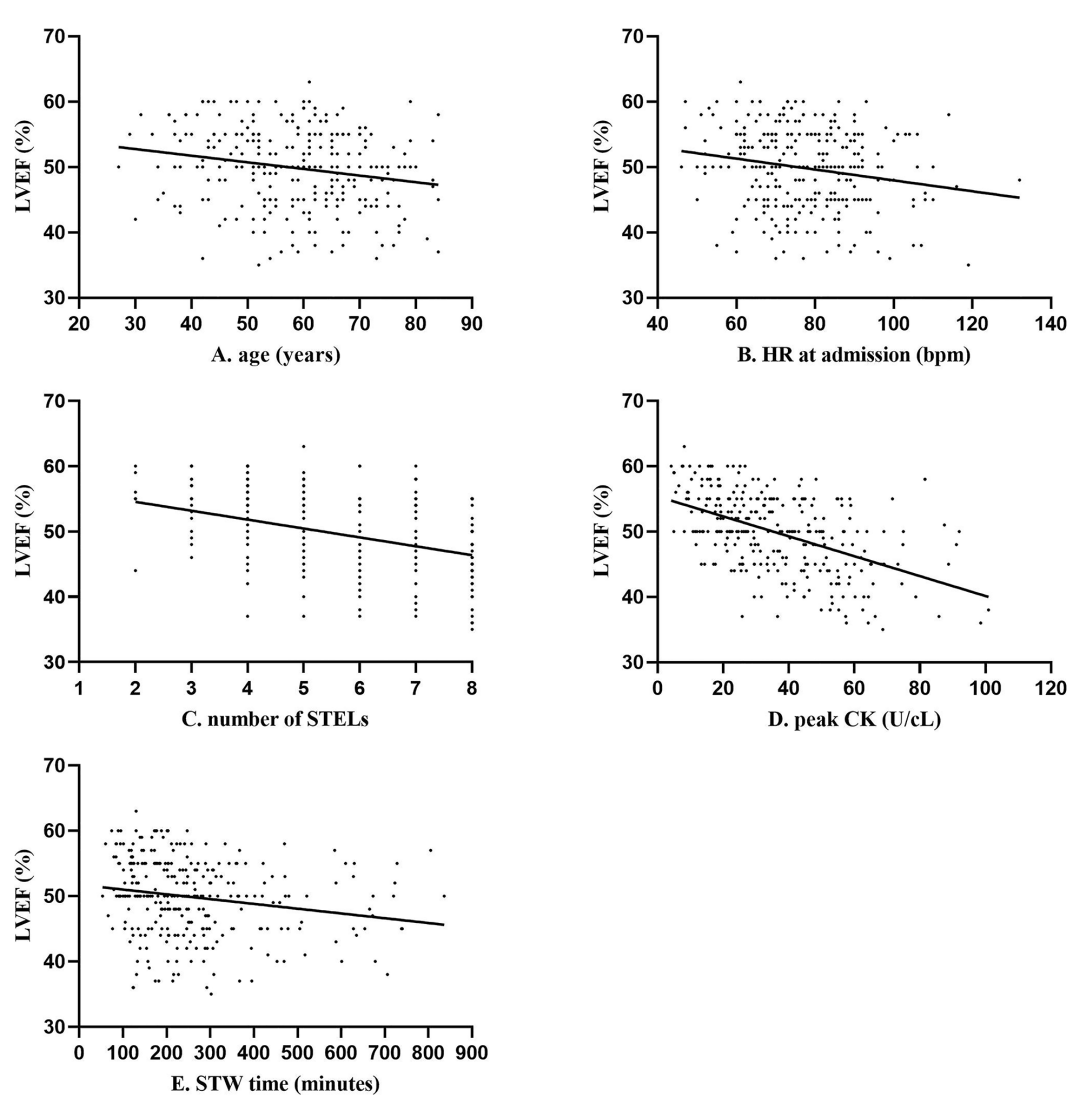
Simple linear regression between LVEF with (A) age, (B) HR at admission, (C) number of STELs, (D) peak CK, (E) STW time, *P* < 0.05

**Fig. 3:**
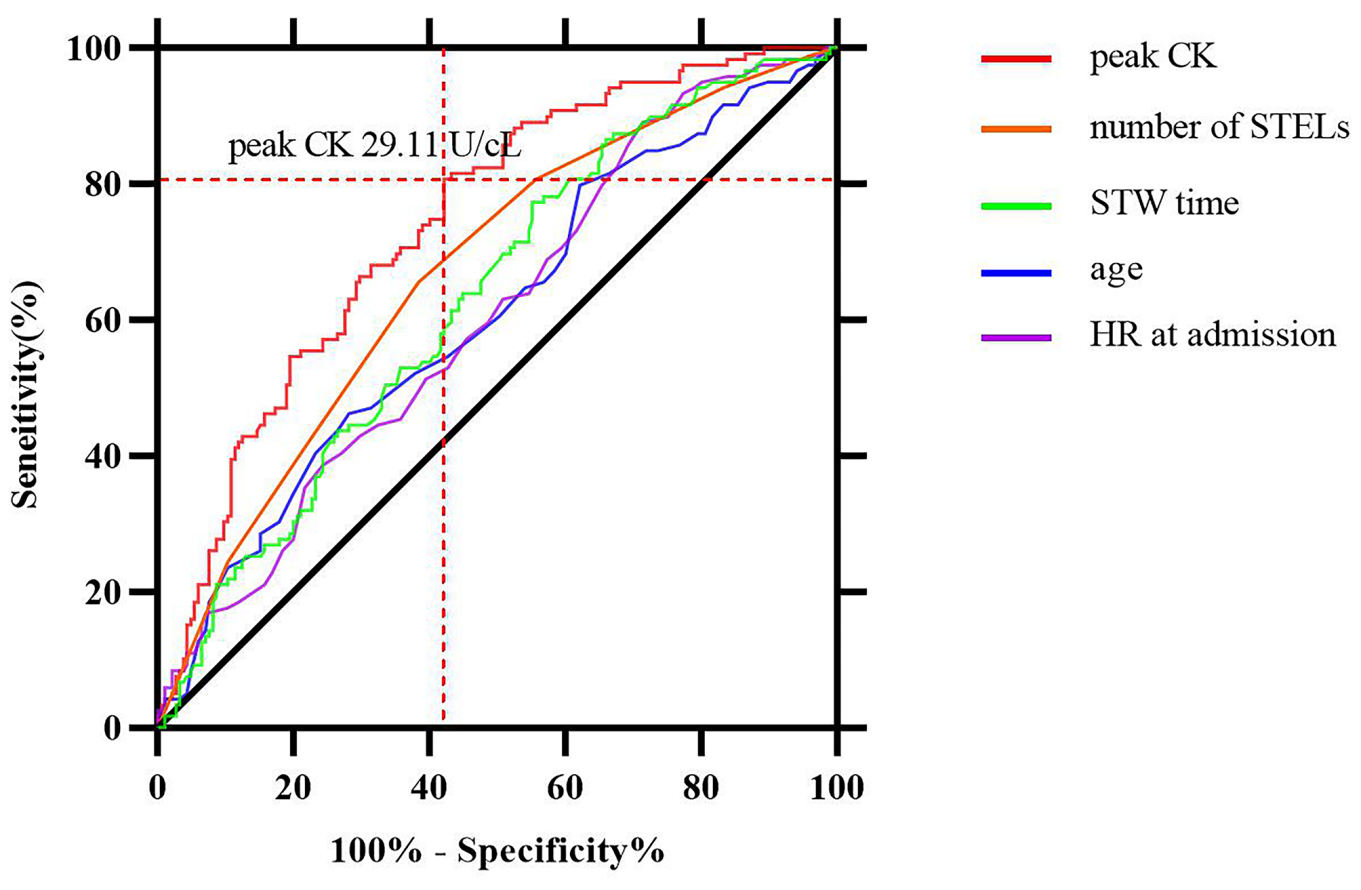
The ROC curve analysis for predicting LVSD

On multivariate analysis, LVSD was used as the dependent variable, and the significantly different indicators on univariate analysis in Table 1 were used as the independent variables. The result showed that age, HR at admission, number of STELs, STW time and peak CK were independent risk factors for LVSD, see Table 2. The simple linear regressions between LVEF and the independent variables are presented in Fig. 2. The ROC analysis showed that the peak CK had the strongest predictive value for LVSD, and the AUC value was 0.742 (CI, 0.687 to 0.797). At a cutoff value of 29.11 U/cL, peak CK exhibited 80.67% diagnostic sensitivity and 57.84% specificity. The AUC values of other related independent variables were less than 0.70, see Fig. 3.

**Table 2:** Multivariate logistic regression analysis of LVSD in patients with AAW-STEMI performed PPCI

Predictors	B	SE	Wals	OR (95%C.I.)	*P*-value
Age	0.057	0.013	19.649	1.058 (1.032,1.085)	<0.001
HR at admission	0.027	0.011	5.862	1.027 (1.005,1.049)	0.015
Number of STELs	0.226	0.090	6.254	1.253 (1.050,1.496)	0.012
Peak CK	0.046	0.009	28.202	1.047 (1.029,1.065)	<0.001
STW time	0.002	0.001	4.362	1.002 (1.000,1.004)	0.037

SE: standard error; OR: odds ratios; CI: confidence interval

In subgroup analysis, patients with HR ≥ 80 bpm at admission were more likely to develop LVSD than patients with HR < 70 bpm (*P* < 0.05). Peak CK was positively correlated with the number of STELs. Opening or proximal occlusion of LAD artery generated a significant increase in CK than middle or distal occlusion (*P* < 0.05). Peak CK was higher in younger AAW-STEMI patients whose age were less than 50 years [41.61(25.08,58.28) vs. 30.66(20.34,47.20) U/cL, *P* < 0.01]. The STW time was approximately 30 min longer in the ≥ 60 years group than in the < 60 years group (*P* < 0.05). The average first medical contact to wire-crossing (FTW) time was 77.11 min, the fastest time was 19 min, the longest time was 237 min, the proportion of patients with less than 90 min was 75.24%, and 37.79% achieved the currently advocated FTW of < 60 min.

**Fig. 4:**
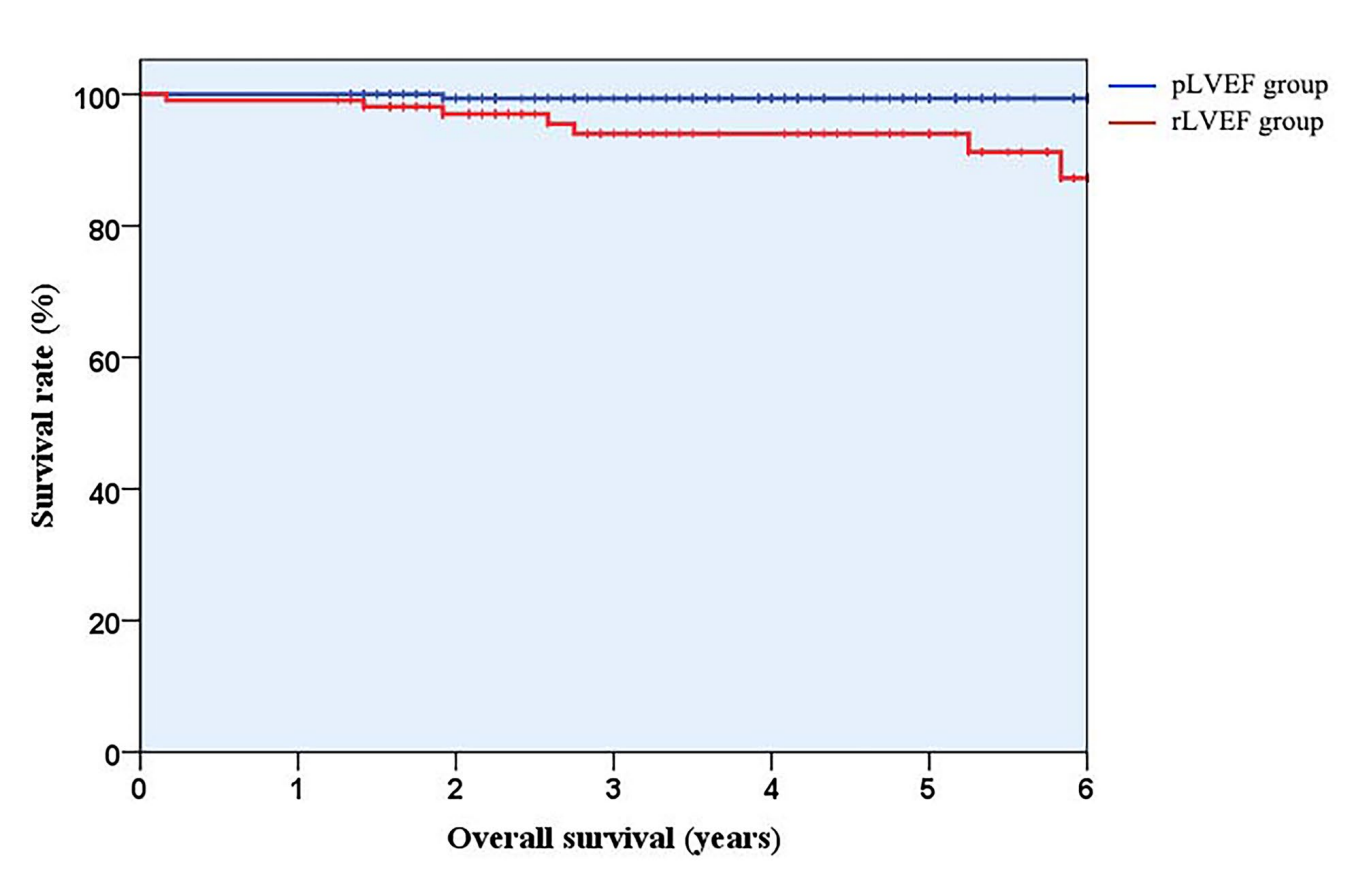
Kaplan-Meier survival curves up to 6-year follow-up

There were 15 patients lost to follow up, the rate was 4.93%. Except for 2 patients with non-cardiovascular death, a total of 8 patients dead of cardiovascular disease after a median follow-up of 47 months (interquartile range: 27 to 64 months): 7 (6.54%) patients in the rLVEF group and 1 (0.56%) patient in the pLVEF group, respectively (hazard ratio: 12.11; 95% confidence interval: 1.49 to 98.70 [*P* = 0.02]). The total number of exposed persons was 821, and the cardiovascular mortality rate was 0.97 per 100 person-years. Univariate Cox regression analysis was performed for each suspected confounding factor. Prior ischemic stroke, GRACE score and rLVEF were adverse predictors for cardiovascular death in patients with AAW-STEMI reperfused by PPCI (*P* < 0.05). These three indicators from univariate analysis were included in multivariate Cox regression analysis, demonstrating that rLVEF was an independent predictor of cardiovascular death, see Table 3. The Kaplan‒Meier survival curves up to 6-year follow-up are shown in Fig. 4.

**Table 3:** Univariate and multivariate Cox hazard analysis of risk factors for future cardiovascular death

	Univariate analysis				Multivariate analysis		
	HR	95%C.I.	*P*-value		HR	95%C.I.	*P*-value
Prior ischemic stroke	0.152	0.038,0.607	0.008		0.312	0.076,1.282	0.106
GRACE score	1.052	1.013,1.094	0.009		1.041	0.998,1.086	0.061
LVEF	0.797	0.700,0.908	0.001		0.818	0.712,0.940	0.005

## Discussion

LVSD is often the consequence of an irreversible loss of viable mass following STEMI, occasionally in combination with loss of contractility in ischemic, but still viable myocardium (hibernating myocardium) [10]. LVEF is one of the most general indicators of LVSD and is widely available in clinical settings. Evaluation of LVEF after STEMI treated with PPCI is well established as an important predictor of clinical outcome [11]. By using the Kaplan‒Meier method, we found significant differences in the survival rate between the rLVEF group and pLVEF group. A lower baseline LVEF and larger infarct size have been associated with failure of LV recovery [12]. Timely identification of subjects at risk for HF development using a multimodality approach and early initiation of guideline-directed HF therapy in these patients can decrease the HF burden [13]. Previous studies had confirmed that age, delayed presentation, previous history of myocardial infarction, atrial fibrillation, hyperlipidaemia, renal impairment, anterior localization of AMI and proximal lesion location were the risk factors of LVSD in STEMI patients [14–18]. And some markers, such as peak CK, B-type natriuretic peptide, fasting blood glucose, stress marker total thiol groups, and fibrosis marker HE-4 had been confirmed to be associated with rLVEF after STEMI [19–21]. In this study, we found that age, HR at admission, number of STELs, peak CK, and STW time may be used to identify the high risk of HF timely for patients with anterior wall STEMI whose LAD artery was completely occluded and reperfused by PPCI. Moreover, these risk factors are common clinical variables which will make the clinical application of the research results more widespread. We will then briefly discuss each of these risk factors, emphasizing the importance of identifying them and initiating early standard therapy.

### Age

The number of individuals who develop HF after AMI undergoing PCI increases with age [22]. In an observational study of elderly patients with first MI, 75% of survivors developed HF in the subsequent 5 years [23]. In young patients, only nearly one-third presented with LV dysfunction post-MI, and LVEF recovery occurred in more than 40% [24]. The following reasons can explain this phenomenon. The mechanical contractility of the heart, function of the conduction system, heart reserve capacity and tolerance to ischemia or hypoxia are reduced in elderly individuals. In addition, the severity and extent of coronary artery disease increase with age. Patients with multivessel coronary artery disease have diminished coronary flow reserve and more severe myocardial damage after AMI. For older patients, we need to pay more attention to the seriousness of STEMI.

### Heart rate at admission

Accelerated HR is an important external manifestation of sympathetic nerve excitement, which is one of the primary internal mechanisms for the occurrence and development of HF after AMI. In the early stage of AMI, increased HR further promotes myocardial oxygen consumption and shortens myocardial perfusion time, which can directly cause the expansion of the infarction size. As the stroke volume decreases and coronary perfusion decreases, HR increases in an attempt to regain cardiac output and maintain sufficient perfusion of the heart [25]. The formation of a vicious cycle leads to a rapid deterioration of heart function. A study showed that prehospital HR ≥ 100 bpm in STEMI was independently associated with larger infarct size, reduced LVEF and an increased risk of all-cause mortality and HF [26]. Therefore, HR at admission serves as an easily obtainable and powerful tool to identify STEMI patients at high risk.

### Number of ST-segment elevation leads

Electrocardiography has several advantages including simplicity, non-invasive and low cost. It not only has the ability to estimate the location and range of a myocardial ischemic area, but also can be used to predict possible adverse events and complications, playing a central role in the diagnosis of STEMI in an emergency setting. Blood is supplied by LAD artery to the left anterior wall, the anterior lateral wall, and the front two-thirds of the ventricular septum. When LAD artery is blocked, those area will be damaged or even necrosis, and the corresponding anterior wall leads will show typical “damage type” changes, namely ST segment elevation. Anterior myocardial infarctions lead to more pronounced LVSD and more adverse LV remodeling compared with myocardial infarction in other areas [27].When occlusion occurs near the proximal of LAD artery, in addition to lesions at the base of left ventricle, the anterior wall, left lateral wall and ventricular septum will also be involved, causing elevation of the ST-segment of anterior (V1 ~ V4) and lateral (I, aVL, and V5 ~ V6) [28, 29]. When the occlusion site is at distal segment of LAD artery, the ST segment elevation is more obvious in V4-V6 leads, but not in V1-V2 or aVL leads [30]. Similarly, the greater the number of ST elevation leads that appear, the wider the myocardial injury and infarct range will be. The number of leads with ST-segment elevation was associated with LV remolding occurrence [31]. For patients especially with extensive anterior wall combined with high sidewall ST-segment elevation, we need to be aware of the risk of their illness.

### Peak creatine kinase

CK has certain value for the early diagnosis and severity of AMI patients. CK mainly exists in skeletal muscle and myocardium, and also exists in brain tissue. It plays an important role in catalyzing cell energy conversion and regulating cell electrophysiological activities. When MI occurs, myocardial cells rupture and necrosis, and then CK will be released into the blood. CK will increase within 6 h, and reach peak at about 24 h, and then return to normal, generally lasting for 3–4 days. A large infarct size is associated with progressive LV remodeling. Patients with higher peak CK are more likely to experience left ventricular remodeling after STEMI and present more often with a lower LVEF [32, 33]. Patients with AAW-STEMI are at increased risk for higher CK [34]. Peak CK was higher in younger patients (< 50 years) in this study. This may be due to decreased protein synthesis and lower levels of CK in the aged cardiomyocytes of elderly individuals [35]. In addition, because of the lack of ischemic preconditioning and less collateral circulation, the tolerance of cardiomyocytes to acute ischemia was reduced in younger patients. Monitoring peak CK is necessary for all patients with STEMI.

### Symptoms to wire-crossing time

Larger infarct size is associated with greater LV dysfunction, adverse cardiac remodeling, and HF over time. Shortening the STW time is very important to reduce the risk of HF in patients, especially those with AAW-STEMI [36]. STW time includes the time from onset of symptoms to first medical contact (STF) and FTW time. Older patients over 60 years had longer STW time than those under 60 years. It is considered that it may be the cognitive and socioeconomic factors of older patients contributing further to the delay. The proportion of FTW time achieved the currently advocated FTW of < 60 min is lower relatively. Several reasons could explain the prolonged FTW time, including the complicated treatment process in the emergency department for STEMI patients during the COVID-19 pandemic, the extremely low ratio of patients admitted directly to the catheterization laboratory, the occupancy of the catheter room and the hesitation of the patient’s family on the PCI operation. STW time is an independent negative influencing factor of LVEF in patients with anterior wall STEMI in the acute phase. It is still necessary to strengthen the publicity of AMI knowledge among the public to emphasize the importance of timely consultation for patients with chest pain. All efforts should be extended to shorten the STW time by educating the public to activate emergency medical services early to bypass the emergency department and allow timely PPCI for the best outcome [36].

### Recommendations for early treatment and follow-up

STEMI is associated with cardiomyocyte loss, fibrosis, and cardiac remodeling, which together represent the main pathophysiological mechanisms that comprise the clinical picture of HF. Timely reperfusion therapy can save the ischemic myocardium and reduce the loss of viable mass. Medication following reperfusion therapy is also critical to improving prognosis. In this study, early application of ACEIs/ARBs or ARNIs and β-blockers failed to improve the LVEF level in the acute phase. However, it has been confirmed that the early application of ACEIs/ARBs or ARNIs and β-blockers can inhibit myocardial remodeling and improve long-term prognosis [37]. Improvement in LVEF commonly begins within 3 days in patients who are revascularized with myocardial stunning and improved function of viable myocardium as the mechanisms, and the main improvement in LVEF occurs within 1 month [38, 39]. The first month following primary reperfusion is a critical period during which the greatest degree of cardiac remodeling occurs [40]. Early prediction of LVSD and timely application of recombinant human brain natriuretic peptide, levosimendan, aldosterone antagonist or sodium-glucose cotransporter type 2 inhibitors may reduce the occurrence of HF after AMI [41–44]. LVSD occurred in more than one-third of patients with AAW-STEMI reperfused by PPCI in our study. Closer follow-up is recommended for patients with LVSD to benefit from early optimization of HF treatment, thereby reducing or delaying cardiogenic death.

### Limitations

Several limitations should be acknowledged. First, the present study was a single-center, retrospective, observational study, and unexpected and unmeasured confounders may have affected the outcomes. Second, we studied patients whose cardiac function was Killip grade 1 because we observed that this population accounted for the majority of patients who presented within 12 hours of the onset of AAW-STEMI. In contrast to the conventional treatment for Killip class 1 cardiac function, treatment for HF was initiated for patients with Killip grade 2 or above upon admission. And we selected patients who had improved after hospitalization and were discharged from the hospital, excluding those who died during hospitalization. Therefore, this patient subgroup was not entirely representative of the overall cohort. Third, in multivariable Cox regression analysis, there were only 8 events of cardiovascular death. Although the number of events was small, the difference in cardiovascular mortality between pLVEF group and rLVEF group was extremely significant. Fourth, LVEF calculated by transthoracic echocardiography was a crude estimation of the true performance. Ideally, cardiac MRI would better define the extent of cardiac damage. Fifth, due to the lack of dynamic measurements of LVEF, we did not analyze and discuss the effect of changes in LVEF on prognosis during follow-up. Sixth, the rate of prescribing optimal drug therapy after STEMI was lower than expected in our cohort. Last, the sample size of the present study might be relatively small, and the proportion of very elderly patients in this study was lower.

## Conclusions

Our findings suggested that age, HR at admission, number of STELs, peak CK, and STW time were independent risk factors for LVSD in the acute phase in patients with AAW-STEMI whose LAD artery was completely occluded and underwent PPCI. HR at admission and STW time are factors that we can intervene in. All these predictors may be used to identify timely the risk of LVSD and initiate early standard therapy in these patients to decrease the occurrence of HF as much as possible. Incident LVSD in the acute phase after AAW-STEMI was significantly associated with long-term adverse outcomes. For patients with LVSD, closer follow-up is recommended to help these patients benefit from early optimization.

## Data Availability

The data will become available immediately following publication and there is no end date. The datasets used in the study are available from the corresponding author on reasonable request.

## Abbreviations

AAW-STEMI: Acute anterior wall ST-segment elevation myocardial infarction
ACEIs: Angiotensin converting-enzyme inhibitors
AMI: Acute myocardial infarction
ARBs: Angiotensin receptor blockers
ARNIs: Angiotensin receptor II blocker-neprilysin inhibitors
AUC: Area under the curve
CK: Creatine kinase
FTW: First medical contact to wire-crossing
GRACE: Global registry of acute coronary events risk
HDL: High-density lipoprotein
HF: Heart failure
HR: Heart rate
IQR: Interquartile range
LAD: Left anterior descending
LV: Left ventricular
LVEF: Left ventricular ejection fraction
LVSD: Left ventricular systolic dysfunction
MI: Myocardial infarction
NT-proBNP: N-terminal pro brain natriuretic peptide
pLVEF: Preserved left ventricular ejection fraction
PPCI: Primary percutaneous coronary intervention
rLVEF: Reduced left ventricular ejection fraction
ROC: Receiver operating characteristic
STELs: ST-segment elevation leads
STF: Symptoms to first medical contact
STW: Symptom to wire-crossing
TIMI: Thrombolysis in myocardial infarction
